# Supplementary material for: Physics-informed transformers for electronic quantum states
Source: Nat Commun. 2025 Nov 28;16:10811. doi: 10.1038/s41467-025-66844-z (PMC12669604; doi:10.1038/s41467-025-66844-z)
Supplement: Supplementary file 1 — Supplementary information [file 41467_2025_66844_MOESM1_ESM.pdf]

Supplementary Information for Physics-Informed Transformer for Electronic Quantum States

João Augusto Sobral,[1] Michael Perle,[2] and Mathias S. Scheurer[1]

[1] *Institute for Theoretical Physics III, University of Stuttgart, 70550 Stuttgart, Germany*
[2] *Institute for Theoretical Physics, University of Innsbruck, A-6020, Innsbruck, Austria*

CONTENTS

Supplementary Note A: Fermionic Model 1
1. General basis 1
2. Expectation values in the chiral basis 2
3. Expectation values in a general basis 4
4. Hartree-Fock implementation for the fermionic model 5

Supplementary Note B: Variational Monte Carlo 8
5. Calculating observables 8
6. Performance analysis of optimizers and hyperparameters 8
7. Further results: Larger system sizes, n_U scaling at the critical region and locality 10

SUPPLEMENTARY NOTE A: FERMIONIC MODEL

1. General basis

The Hamiltonian from Equation (7) can also be naturally represented in a general basis as

$$\hat{H} = t \sum_{\boldsymbol{k}\in\mathrm{BZ}} \cos(\boldsymbol{k})\, \bar{d}^{\dagger}_{\boldsymbol{k},\alpha} \mathcal{P}_{\alpha,\beta}(\boldsymbol{k})\, \bar{d}_{\boldsymbol{k},\beta} + U \sum_{\boldsymbol{q}\in\mathbb{R}} V(\boldsymbol{q}) \rho_{\boldsymbol{q}} \rho_{-\boldsymbol{q}}, \tag{1}$$

with the density operator

$$\rho_{\boldsymbol{q}} = \sum_{k\in\mathrm{BZ}} \left(\bar{d}^{\dagger}_{\mathrm{BZ}(\boldsymbol{k}+\boldsymbol{q}),\alpha} \mathcal{F}_{\alpha,\beta}(\boldsymbol{k}, q)\, \bar{d}_{\boldsymbol{k},\beta} - \sum_{\boldsymbol{G}\in\mathrm{RL}} \delta_{q,G} f_1(\boldsymbol{k}, \boldsymbol{G}) \right), \tag{2}$$

where BZ indicates the Brillouin Zone defined as $[-\pi, \ldots, \pi - 2\pi/N_{\boldsymbol{k}}]$. The form factors are given by

$$\mathcal{P}_{\alpha,\beta}(\boldsymbol{k}) = \left(U^{\dagger}_{\boldsymbol{k}} \sigma_x U_{\boldsymbol{k}} \right)_{\alpha,\beta} \tag{3}$$

and

$$\mathcal{F}_{\alpha,\beta}(\boldsymbol{k}, \boldsymbol{q}) = \sum_{\gamma=\pm} \left(U^{\dagger}_{\boldsymbol{k}} \right)_{\alpha,\gamma} \left[f_1(\boldsymbol{k}, \boldsymbol{q}) + i\gamma f_2(\boldsymbol{k}, \boldsymbol{q}) \right] \left(U_{\boldsymbol{k}} \right)_{\gamma,\beta}. \tag{4}$$

The expressions $f_j(\boldsymbol{k}, \boldsymbol{q})$ in Supplementary Equation (4) can be further determined based on the desired symmetries for the model. Since $\rho_{\boldsymbol{q}}$ is the Fourier transform of the density operator, it needs to obey $\rho^{\dagger}_{\boldsymbol{q}} = \rho_{-\boldsymbol{q}}$. We impose C_{2z} invariance, which leads to the following constraints on the form factors

$$\begin{aligned} f_1(\boldsymbol{k}, \boldsymbol{q}) &= f_1(\mathrm{BZ}(\boldsymbol{k}+\boldsymbol{q}), -\boldsymbol{q}) \\ f_2(\boldsymbol{k}, \boldsymbol{q}) &= -f_2(\mathrm{BZ}(\boldsymbol{k}+\boldsymbol{q}), -\boldsymbol{q}) \\ f_j(\boldsymbol{k}, \boldsymbol{q}) &= f_j(-\boldsymbol{k}, -\boldsymbol{q}). \end{aligned} \tag{5}$$

In turn, these imply that

$$\begin{aligned} f_1(\boldsymbol{k}, \boldsymbol{G}) &= f_1(-\boldsymbol{k}, \boldsymbol{G}), \\ f_2(\boldsymbol{k}, \boldsymbol{G}) &= -f_2(-\boldsymbol{k}, \boldsymbol{G}), \quad \forall \boldsymbol{G} \in \mathrm{RL}. \end{aligned} \tag{6}$$

A possible choice that satisfies Supplementary Equations (5) and (6) is given by

$$f_j\left(k,q\right)=\begin{cases}1, \text{ for } j=1\\ \beta\sin\left(k\right)\left(\sin\left(q\right)+\sin\left(k+q\right)\right), \text{ for } j=2\end{cases}. \tag{7}$$

Here, β mediates the strength difference between both form factors. Without loss of generality, we set $\beta = 0.9$.

The chiral (interacting limit) and band (kinetic limit) bases can be retrieved from Supplementary Equations (1)-(4) as follows. For $t/U \to 0$, we can consider the states

$$|\pm\rangle = \prod_{\boldsymbol{k}\in\mathrm{BZ}} \bar{d}^\dagger_{\boldsymbol{k},\pm}|0\rangle, \tag{8}$$

where $\bar{d}^\dagger_{\boldsymbol{k},\pm}$ are the creation operators defined with $U_{\boldsymbol{k}} = \mathbb{I}$ in Supplementary Equation (1). The Hamiltonian takes a positive semi-definite form $\hat{H} \propto \sum_{\boldsymbol{q}\in\mathbb{R}} V(\boldsymbol{q})\rho_{\boldsymbol{q}}\rho^\dagger_{\boldsymbol{q}}$. Given that the states defined in Supplementary Equation (8) obey $\rho_{\boldsymbol{q}}|\psi_\pm\rangle = 0$, we conclude that they represent the ground state of the system, and thus, the model becomes exactly solvable in this limit.

Similarly, the *band*-basis, defined with $U_{\boldsymbol{k}} = \frac{1}{\sqrt{2}}\begin{pmatrix}1 & -i\\ 1 & i\end{pmatrix}$, in Supplementary Equation (1), acquires the diagonal form $\hat{H} \propto \sum_{\boldsymbol{k}\in\mathrm{BZ}}\cos(\boldsymbol{k})d^\dagger_{\boldsymbol{k}}\sigma_z d_{\boldsymbol{k}} = \sum_{\boldsymbol{k}\in\mathrm{BZ}}\sum_{j=1,2}\varepsilon_{\boldsymbol{k},j}d^\dagger_{\boldsymbol{k},j}d_{\boldsymbol{k},j}$ in band space with $\varepsilon_{\boldsymbol{k},j} = (-1)^j\cos\boldsymbol{k}$ for large t/U. If the total number of electrons N_e is chosen such that $\{-\frac{\pi}{2},\frac{\pi}{2}\} \notin$ BZ, the ground state is obtained by filling the lower band as

$$|\psi_-\rangle = \prod_{\boldsymbol{k}\in\mathrm{BZ}} d^\dagger_{\boldsymbol{k},-}|0\rangle \quad \text{where} \quad d^\dagger_{\boldsymbol{k},-} = \begin{cases} d^\dagger_{\boldsymbol{k},1} & |\boldsymbol{k}| < \frac{\pi}{2}\\ d^\dagger_{\boldsymbol{k},0} & |\boldsymbol{k}| > \frac{\pi}{2}\end{cases}. \tag{9}$$

For finite t/U, an optimal basis choice becomes non-trivial. Some intuition can be acquired when expressing the kinetic term in the chiral basis, which yields $\hat{H} = \sum_{\boldsymbol{k}\in\mathrm{BZ}}\cos(\boldsymbol{k})\bar{d}^\dagger_{\boldsymbol{k}}\sigma_x\bar{d}_{\boldsymbol{k}}$. This structure indicates that within second-order perturbation theory, other states $\boldsymbol{s}$ will be admixed with the states defined in Supplementary Equation (8), where some $d^\dagger_{\boldsymbol{k}\pm}$ will be replaced with $d^\dagger_{\boldsymbol{k}\mp}$. Despite this mixing, the $|\psi_\pm\rangle$ will remain degenerate since the Hamiltonian is invariant under the $\boldsymbol{k}$-local anti-unitary symmetry described by the anti-unitary operator PT with $PTd_{\boldsymbol{k}}(PT)^\dagger = \sigma_x d_{\boldsymbol{k}}$ and $PT|\psi_+\rangle \propto |\psi_-\rangle$, where T indicates the time reversal symmetry operator.

2. Expectation values in the chiral basis

For the evaluation of the energy expectation value, it is instructive to start with a fixed basis. We choose the chiral basis, and then generalize it to an arbitrary basis. For notational simplicity, we omit explicit mention of the $|\mathrm{RS}\rangle$ state removal in the following considerations, as they remain valid whether or not we include it. The variational ansatz can be written as

$$|\psi_{\boldsymbol{\theta}}\rangle = \sum_{\boldsymbol{s}\in[+,-]^{N_e}} \psi_{\boldsymbol{\theta}}(\boldsymbol{s})\prod_k \bar{d}^\dagger_{k,(\boldsymbol{s})_k}|0\rangle. \tag{10}$$

For $t/U = 0$ the ground state has $\psi_{\boldsymbol{\theta}}(\boldsymbol{s}) = 0$ except for $\boldsymbol{s} = (+,+,+,\ldots,+)$ (or all $-$). Finite t will admix other $\boldsymbol{s}$ configurations.

To calculate the expectation value of $\hat{H}$, we need the matrix elements

$$H_{\boldsymbol{s}\boldsymbol{s}'} := \langle\boldsymbol{s}|H|\boldsymbol{s}'\rangle, \tag{11}$$

where $|\boldsymbol{s}\rangle$ are the basis states. Without loss of generality, we consider the band mapping $s_i = 0,1 \to -1,1(-,+)$ for what follows. Additionally, we introduce the notation

$$[\boldsymbol{s}]_k = \begin{cases}(s)_{k'} & \text{for } k\neq k'\\ -(s)_k & \text{for } k = k'\end{cases} \tag{12}$$

to indicate a state which is equal to $\boldsymbol{s}$ apart from the hopping of one electron to another band at the k-position. Let us begin with the non-interacting (kinetic) part $\hat{H}_0$ of the Hamiltonian:

$$\langle\boldsymbol{s}|\hat{H}_0|\boldsymbol{s}'\rangle = t\sum_{\boldsymbol{k}}\cos(\boldsymbol{k})\delta_{[\boldsymbol{s}]_{\boldsymbol{k}}\boldsymbol{s}'}. \tag{13}$$

The associated "local Hamiltonian" then simply reads as

$$\hat{H}^0_{\rm loc}(\boldsymbol{s}) = t\sum_{\boldsymbol{k}} \cos(\boldsymbol{k})\frac{\psi_{\boldsymbol{\theta}}([\boldsymbol{s}]_k)}{\psi_{\boldsymbol{\theta}}(\boldsymbol{s})}, \tag{14}$$

according to Supplementary Equation (13). If we only have this non-interacting part (or asymptotically in the limit $|t/U| \gg 1$), we will therefore have

$$\psi_{\boldsymbol{\theta}}(\boldsymbol{s}) = \prod_k \frac{1}{\sqrt{2}}\left(\delta_{(\boldsymbol{s})_k,+} - \text{sign}(t\cos k)\delta_{(\boldsymbol{s})_k,-}\right) \tag{15}$$

in the ground state (with associated energy $E = -t\sum_{\boldsymbol{k}}|\cos\boldsymbol{k}|$). The momentum-resolved fermionic bilinears defined in Equation (11) can be calculated in a similar manner. For the x-component, for example

$$\langle \boldsymbol{s}|\mathcal{N}^x_{\boldsymbol{k}}|\boldsymbol{s}'\rangle = \delta_{[\boldsymbol{s}]_k,\boldsymbol{s}'} \quad \text{with} \quad \mathcal{N}^{x,\rm loc}_k(\boldsymbol{s}) = \frac{\psi_{\boldsymbol{\theta}}([\boldsymbol{s}]_k)}{\psi_{\boldsymbol{\theta}}(\boldsymbol{s})}. \tag{16}$$

Now we focus on the interaction part, i.e., $\hat{H}_1 = \sum_{\boldsymbol{q}\in\mathbb{R}} V(\boldsymbol{q})\rho_{\boldsymbol{q}}\rho_{-\boldsymbol{q}}$. To compute the matrix elements $\langle\boldsymbol{s}|H_1|\boldsymbol{s}'\rangle$, we consider the expressions

$$\langle\boldsymbol{s}|\bar{d}^\dagger_{\boldsymbol{k}+\boldsymbol{q},p}\bar{d}_{\boldsymbol{k},p}|\boldsymbol{s}'\rangle = \delta_{\boldsymbol{q}\in\rm RL}\delta_{\boldsymbol{s},\boldsymbol{s}'}\delta_{(\boldsymbol{s})_k,p}, \tag{17}$$

and

$$\begin{aligned}\langle\boldsymbol{s}|\bar{d}^\dagger_{\boldsymbol{k}+\boldsymbol{q},p}\bar{d}_{\boldsymbol{k},p}\bar{d}^\dagger_{\boldsymbol{k}'-\boldsymbol{q},p'}\bar{d}_{\boldsymbol{k}',p'}|\boldsymbol{s}'\rangle &= \delta_{\boldsymbol{q}\in\rm RL}\delta_{\boldsymbol{s},\boldsymbol{s}'}\delta_{(\boldsymbol{s})_k,p}\delta_{(\boldsymbol{s})_{k'},p'} + \\ &+ \delta_{\boldsymbol{q}\notin\rm RL}\delta_{\boldsymbol{k},{\rm BZ}(\boldsymbol{k}'-\boldsymbol{q})}\delta_{(\boldsymbol{s}')_{k'},p'}\delta_{(\boldsymbol{s}')_{k'-q},-p'}\delta_{(\boldsymbol{s})_{k'-q},-p}\delta_{(\boldsymbol{s})_{k'},p}\left(\prod_{k_1\neq k',k'-q}\delta_{(\boldsymbol{s})_{k_1},(\boldsymbol{s}')_{k_1}}\right)\left[\delta_{p,p'} - \delta_{p,-p'}\right].\end{aligned} \tag{18}$$

Here, we introduced $\delta_{\boldsymbol{q}\in\rm RL} := \sum_{\boldsymbol{G}\in\rm RL}\delta_{\boldsymbol{q},\boldsymbol{G}}$ and $\delta_{\boldsymbol{q}\notin\rm RL} := 1 - \delta_{\boldsymbol{q}\in\rm RL}$. The fact that only a small subset of matrix elements is non-zero follows from the restriction to states without double occupancy. Sticking to the current approximation, we end up with the following form of the matrix elements

$$\begin{aligned}\langle\boldsymbol{s}|\hat{H}_1|\boldsymbol{s}'\rangle = \delta_{\boldsymbol{s},\boldsymbol{s}'}h_1(\boldsymbol{s}) + \sum_{\boldsymbol{q}\in\mathbb{R}\backslash\rm RL} V(\boldsymbol{q})\sum_{\boldsymbol{k}\in\rm BZ}\sum_{p,p'} f^*_p(\boldsymbol{k},-\boldsymbol{q})f_{p'}(\boldsymbol{k},-\boldsymbol{q})\left[\delta_{p,p'} - \delta_{p,-p'}\right] \\ \times\,\delta_{(\boldsymbol{s}')_{k'},p'}\delta_{(\boldsymbol{s}')_{k'-q},-p'}\delta_{(\boldsymbol{s})_{k'-q},-p}\delta_{(\boldsymbol{s})_{k'},p}\left(\prod_{k_1\neq k',k'-q}\delta_{(\boldsymbol{s})_{k_1},(\boldsymbol{s}')_{k_1}}\right),\end{aligned} \tag{19}$$

where $f_p(\boldsymbol{k},\boldsymbol{q}) = f_1(\boldsymbol{k},\boldsymbol{q}) + ipf_2(\boldsymbol{k},\boldsymbol{q})$ and the diagonal matrix elements are given by

$$h_1(\boldsymbol{s}) = \sum_{\boldsymbol{G}\in\rm RL} V(\boldsymbol{G})\sum_{\boldsymbol{k},\boldsymbol{k}'}\Bigg[f_1(\boldsymbol{k},\boldsymbol{G})f_1(\boldsymbol{k}',-\boldsymbol{G}) - \sum_{r=\pm} f_1(\boldsymbol{k},r\boldsymbol{G})\sum_p f_p(\boldsymbol{k}',-r\boldsymbol{G})\delta_{(\boldsymbol{s})_{k'},p} \tag{20}$$

$$+\sum_{p,p'} f_p(\boldsymbol{k},\boldsymbol{G})f_{p'}(\boldsymbol{k}',-\boldsymbol{G})\delta_{(\boldsymbol{s})_k,p}\delta_{(\boldsymbol{s})_{k'},p'}\Bigg]. \tag{21}$$

As a simple consistency check, one can see from these expressions that $\langle\boldsymbol{s}|\hat{H}_1|(p_0,p_0,\dots,p_0)\rangle = 0$ for any $\boldsymbol{s}$ and $p_0 = \pm$, as it should be [recall Supplementary Equation (8)].

More generally, due to (i) the conditions imposed on the form factors in Supplementary Equations (5) and (6), and (ii) that double occupancy is neglected, we can show that terms proportional to $\delta_{\boldsymbol{q}\in\rm RL}$ do not contribute to the ground state energy, i.e., that $\hat{H}^{\boldsymbol{G}}_1 = \sum_{\boldsymbol{G}\in\rm RL} V(\boldsymbol{G})\rho_{\boldsymbol{G}}\rho^\dagger_{\boldsymbol{G}} = 0$, or equivalently, that all matrix elements $\langle\boldsymbol{s}|\hat{H}^{\boldsymbol{G}}_1|\boldsymbol{s}'\rangle = 0$. From,

$$\rho_{\boldsymbol{G}} = \sum_{\boldsymbol{k}\in\rm BZ}\left(\sum_{p=\pm}\bar{d}^\dagger_{\boldsymbol{k},p}f_1(\boldsymbol{k},\boldsymbol{G})\,\bar{d}_{\boldsymbol{k},p} - f_1(\boldsymbol{k},\boldsymbol{G})\right) \tag{22}$$

we start by decomposing the matrix elements in

$$\langle \boldsymbol{s}|\hat{H}_1^{\boldsymbol{G}}|\boldsymbol{s}'\rangle = \sum_{\boldsymbol{G}\in\mathrm{RL}} V(\boldsymbol{G}) \sum_{\boldsymbol{k},\boldsymbol{k}'\in\mathrm{BZ}} f_1(\boldsymbol{k},\boldsymbol{G}) f_1(\boldsymbol{k}',\boldsymbol{G}) \left(-2\sum_{p=\pm} \langle \boldsymbol{s}|\bar{d}^\dagger_{\boldsymbol{k},p}\bar{d}_{\boldsymbol{k},p}|\boldsymbol{s}'\rangle + \sum_{p,p'=\pm} \langle \boldsymbol{s}|\bar{d}^\dagger_{\boldsymbol{k},p}\bar{d}_{\boldsymbol{k},p}\bar{d}^\dagger_{\boldsymbol{k}',p'}\bar{d}_{\boldsymbol{k}',p'}|\boldsymbol{s}'\rangle + \langle \boldsymbol{s} \mid \boldsymbol{s}'\rangle \right), \tag{23}$$

where

$$\begin{aligned} \langle \boldsymbol{s}|\bar{d}^\dagger_{\boldsymbol{k},p}\bar{d}_{\boldsymbol{k},p}|\boldsymbol{s}'\rangle &= \delta_{(\boldsymbol{s}')_k,p'}\delta_{(\boldsymbol{s})_k,p}\delta_{\boldsymbol{s},\boldsymbol{s}'} = \delta_{(\boldsymbol{s})_k,p}\delta_{\boldsymbol{s},\boldsymbol{s}'} \\ \langle \boldsymbol{s}|\bar{d}^\dagger_{\boldsymbol{k},p}\bar{d}_{\boldsymbol{k},p}\bar{d}^\dagger_{\boldsymbol{k}',p'}\bar{d}_{\boldsymbol{k}',p'}|\boldsymbol{s}'\rangle &= \delta_{(\boldsymbol{s}')_{k'},p'}\delta_{(\boldsymbol{s})_k,p}\delta_{\boldsymbol{s},\boldsymbol{s}'} \end{aligned} \tag{24}$$

leads to

$$\langle \boldsymbol{s}|\hat{H}_1^{\boldsymbol{G}}|\boldsymbol{s}'\rangle = \delta_{\boldsymbol{s},\boldsymbol{s}'} \sum_{\boldsymbol{G}\in\mathrm{RL}} V(\boldsymbol{G}) \sum_{\boldsymbol{k},\boldsymbol{k}'\in\mathrm{BZ}} f_1(\boldsymbol{k},\boldsymbol{G}) f_1(\boldsymbol{k}',\boldsymbol{G}) \left(-2\sum_{p=\pm} \delta_{(\boldsymbol{s})_k,p} + \sum_{p,p'=\pm} \delta_{(\boldsymbol{s}')_{k'},p'}\delta_{(\boldsymbol{s})_k,p} + 1 \right). \tag{25}$$

The expression in parentheses is zero for any combination of $\boldsymbol{s}$ and $\boldsymbol{s}'$ if double occupancy is neglected.

3. Expectation values in a general basis

When working in a general basis, as defined by the transformation in Equation (2) with U_k determined from HF, the new variational ansatz is given as

$$|\psi_{\boldsymbol{\theta}}\rangle = \sum_{\boldsymbol{s}\in[+,-]^{N_e}} \psi_{\boldsymbol{\theta}}(\boldsymbol{s}) \prod_k \bar{d}^\dagger_{\boldsymbol{k},(\boldsymbol{s})_k} |0\rangle . \tag{26}$$

In this case, we need the modified matrix elements

$$\hat{\bar{H}}_{\boldsymbol{s}\boldsymbol{s}'} := \langle \bar{\boldsymbol{s}}|\hat{H}|\bar{\boldsymbol{s}}'\rangle \quad \text{with} \quad |\bar{\boldsymbol{s}}\rangle := \prod_k \bar{d}^\dagger_{\boldsymbol{k},(\boldsymbol{s})_k}|0\rangle , \tag{27}$$

according to Supplementary Equations (1)-(4). The computation of the matrix elements in Supplementary Equation (27) closely parallels that of the previous subsection. For the non-interacting part, we obtain

$$\langle \boldsymbol{s}|\hat{H}_0|\boldsymbol{s}'\rangle = t\sum_{\boldsymbol{k}} \cos(\boldsymbol{k}) \left[\delta_{\boldsymbol{s},\boldsymbol{s}'} \sum_{\alpha=\pm} \mathcal{P}_{\alpha,\alpha}(\boldsymbol{k})\delta_{(\boldsymbol{s})_k,\alpha} + \delta_{[\boldsymbol{s}]_k,\boldsymbol{s}'} \sum_{\alpha=\pm} \mathcal{P}_{\alpha,-\alpha}(\boldsymbol{k})\delta_{(\boldsymbol{s})_k,\alpha} \right], \tag{28}$$

which allows us to identify the local non-interacting energy as

$$\hat{H}^0_{\mathrm{loc}}(\boldsymbol{s}) = t\sum_{\boldsymbol{k}} \cos(\boldsymbol{k}) \left[\mathcal{P}_{\alpha,\alpha}(\boldsymbol{k}) + \mathcal{P}_{\alpha,-\alpha}(\boldsymbol{k}) \frac{\psi_{\boldsymbol{\theta}}([\boldsymbol{s}]_k)}{\psi_{\boldsymbol{\theta}}(\boldsymbol{s})} \right] \quad \text{with} \quad \alpha = (\boldsymbol{s})_k. \tag{29}$$

To write down the matrix elements $\langle \boldsymbol{s}|\hat{H}_1|\boldsymbol{s}'\rangle$ of the interacting part, we first compute the matrix elements

$$\langle \boldsymbol{s}|\bar{d}^\dagger_{\boldsymbol{k}+\boldsymbol{q},\alpha}\bar{d}_{\boldsymbol{k},\beta}|\boldsymbol{s}'\rangle = \delta_{\boldsymbol{q}\in\mathrm{RL}} \left[\delta_{\alpha,\beta}\delta_{\boldsymbol{s},\boldsymbol{s}'}\delta_{(\boldsymbol{s})_k,\alpha} + \delta_{\alpha,-\beta}\delta_{[\boldsymbol{s}]_k,\boldsymbol{s}'}\delta_{(\boldsymbol{s})_k,\alpha} \right], \tag{30}$$

and

$$\begin{aligned} &\langle \boldsymbol{s}|\bar{d}^\dagger_{\boldsymbol{k}+\boldsymbol{q},\alpha}\bar{d}_{\boldsymbol{k},\beta}\bar{d}^\dagger_{\boldsymbol{k}'-\boldsymbol{q},\gamma}\bar{d}_{\boldsymbol{k}',\delta}|\boldsymbol{s}'\rangle \\ &\quad = \delta_{\boldsymbol{q}\in\mathrm{RL}} \left[\delta_{\boldsymbol{k}\neq\boldsymbol{k}'}\delta_{(\boldsymbol{s}')_{k'},\delta}\delta_{(\boldsymbol{s})_{k'},\gamma}\delta_{(\boldsymbol{s}')_k,\beta}\delta_{(\boldsymbol{s})_k,\alpha} + \delta_{\boldsymbol{k},\boldsymbol{k}'}\delta_{\beta,\gamma}\delta_{(\boldsymbol{s})_k,\alpha}\delta_{(\boldsymbol{s}')_k,\delta} \right] \left(\prod_{k_1\neq k',k'-q} \delta_{(\boldsymbol{s})_{k_1},(\boldsymbol{s}')_{k_1}} \right) \\ &\quad + \delta_{\boldsymbol{q}\notin\mathrm{RL}}\delta_{\boldsymbol{k},\mathrm{BZ}(\boldsymbol{k}'-\boldsymbol{q})}\delta_{(\boldsymbol{s}')_{k'},\delta}\delta_{(\boldsymbol{s}')_{k'-q},-\gamma}\delta_{(\boldsymbol{s})_{k'-q},-\beta}\delta_{(\boldsymbol{s})_{k'},\alpha} \left(\prod_{k_1\neq k',k'-q} \delta_{(\boldsymbol{s})_{k_1},(\boldsymbol{s}')_{k_1}} \right) \left[\delta_{\beta,\gamma} - \delta_{\beta,-\gamma} \right]. \end{aligned} \tag{31}$$

With this, the evaluation of $\langle \boldsymbol{s}|\hat{H}_1|\boldsymbol{s'}\rangle$ can be performed for any unitary basis according to Supplementary Equation (2). As another consistency check, we reproduce Supplementary Equations (17) and (18) of the previous section in the limit $\alpha = \beta = p$ and $\gamma = \delta = p'$.

From this consideration, the local interacting contribution to the ground state energy is given by

$$\hat{H}^1_{\text{loc}} = \sum_{\boldsymbol{q}\notin \text{RL}} V(\boldsymbol{q}) \sum_{\boldsymbol{k}\in \text{BZ}} \sum_{\delta\in\{+,-\}} \mathcal{F}_{\alpha,\beta}(\text{BZ}(\boldsymbol{k}-\boldsymbol{q}),\boldsymbol{q}) \times \left[\mathcal{F}_{\beta,\delta}(\boldsymbol{k},-\boldsymbol{q}) \frac{\psi_{\boldsymbol{\theta}}\left([s]_{\boldsymbol{k}\to\delta}\right)}{\psi_{\boldsymbol{\theta}}(s)} - \mathcal{F}_{-\beta,\delta}(\boldsymbol{k},-\boldsymbol{q}) \frac{\psi_{\boldsymbol{\theta}}\left([\boldsymbol{s}]_{k\to\delta;\text{BZ}(k-q)}\right)}{\psi_{\boldsymbol{\theta}}(\boldsymbol{s})} \right], \tag{32}$$

with $\alpha = (\boldsymbol{s})_k$ and $\beta = -(\boldsymbol{s})_{\text{BZ}(k-q)}$. Following the notation introduced in Supplementary Equation (12), we also define

$$[\boldsymbol{s}]_{k\to\delta} = \begin{cases} (s)_{k'} & \text{for } k \neq k' \\ \delta & \text{for } k = k' \end{cases}, \quad \text{and} \quad [\boldsymbol{s}]_{k\to\delta;l} = \begin{cases} (s)_{k'} & \text{for } k \neq k' \text{ and } k' \neq l \\ \delta & \text{for } k = k' \\ -(s)_l & \text{for } k = l \end{cases}. \tag{33}$$

Finally, Supplementary Equation (16) now reads

$$\mathcal{N}^{\text{loc}}_{\boldsymbol{k}}(\boldsymbol{s}) = \mathcal{P}_{\alpha,\alpha}(\boldsymbol{k}) + \mathcal{P}_{\alpha,-\alpha}(\boldsymbol{k}) \frac{\psi_{\boldsymbol{\theta}}\left([\boldsymbol{s}]_k\right)}{\psi_{\boldsymbol{\theta}}(\boldsymbol{s})} \quad \text{with} \quad \alpha = (\boldsymbol{s})_k. \tag{34}$$

4. Hartree-Fock implementation for the fermionic model

Now we show how to obtain the self-consistent HF equations to the Hamiltonian defined in Equation (1), following Christos et al.[1] Starting from the chiral basis, without loss of generality, we note that the kinetic term is already equivalent to its mean-field expression

$$H_0^{\text{HF}} = \sum_{\boldsymbol{k}\in\text{BZ}} d^\dagger_{\boldsymbol{k}} \widetilde{h_{\boldsymbol{k}}} d_{\boldsymbol{k}} \quad \text{with} \quad \widetilde{h_{\boldsymbol{k}}} = t\cos(\boldsymbol{k})\sigma_x. \tag{35}$$

For the interacting term we start from

$$\hat{H}_1 = \sum_{\boldsymbol{q}\notin\text{RL}} V(\boldsymbol{q})\rho_{\boldsymbol{q}}\rho_{-\boldsymbol{q}} = \sum_{\boldsymbol{q}\notin\text{RL}} V(\boldsymbol{q}) \sum_{\boldsymbol{k},\boldsymbol{k'}\in\text{BZ}} d^\dagger_{\text{BZ}(\boldsymbol{k}+\boldsymbol{q}),\alpha} d_{\boldsymbol{k},\beta} d^\dagger_{\text{BZ}(\boldsymbol{k'}-\boldsymbol{q}),\gamma} d_{\boldsymbol{k'},\delta} \mathcal{F}_{\alpha,\beta}(\boldsymbol{k},\boldsymbol{q})\ \mathcal{F}_{\gamma,\delta}(\boldsymbol{k'},-\boldsymbol{q})\ . \tag{36}$$

This can be brought to normal order by using the fermionic anticommutation relation $\{d_{\boldsymbol{k}\beta}, d^\dagger_{\text{BZ}(\boldsymbol{k'}-\boldsymbol{q}),\gamma}\} = \delta_{\boldsymbol{k},\text{BZ}(\boldsymbol{k'}-\boldsymbol{q})}\delta_{\beta,\gamma}$, as

$$\begin{aligned} \hat{H}_1 = &- \sum_{\boldsymbol{q}\notin\text{RL}} V(\boldsymbol{q}) \sum_{\boldsymbol{k},\boldsymbol{k'}\in\text{BZ}} d^\dagger_{\text{BZ}(\boldsymbol{k}+\boldsymbol{q}),\alpha} d^\dagger_{\text{BZ}(\boldsymbol{k'}-\boldsymbol{q}),\gamma} d_{\boldsymbol{k},\beta} d_{\boldsymbol{k'},\delta} \mathcal{F}_{\alpha\beta}(\boldsymbol{k},\boldsymbol{q})\ \mathcal{F}_{\gamma\delta}(\boldsymbol{k'},-\boldsymbol{q})\ + \\ &+ \sum_{\boldsymbol{q}\notin\text{RL}} V(\boldsymbol{q}) \sum_{\boldsymbol{k},\boldsymbol{k'}\in\text{BZ}} d^\dagger_{\text{BZ}(\boldsymbol{k}+\boldsymbol{q}),\alpha} d_{\boldsymbol{k'}\delta} \mathcal{F}_{\alpha\beta}(\boldsymbol{k},\boldsymbol{q})\ \mathcal{F}_{\gamma\delta}(\boldsymbol{k'},-\boldsymbol{q})\ \delta_{\boldsymbol{k},\text{BZ}(\boldsymbol{k'}-\boldsymbol{q})}\delta_{\beta,\gamma}. \end{aligned} \tag{37}$$

The single-body term can be further simplified by first shifting the index $\boldsymbol{k} \to \text{BZ}(\boldsymbol{k}-\boldsymbol{q})$ and $\boldsymbol{q} \to -\boldsymbol{q}$ in

$$\begin{aligned} \hat{H}^{\text{single}}_1 &= \sum_{\boldsymbol{q}\notin\text{RL}} V(\boldsymbol{q}) \sum_{\boldsymbol{k},\boldsymbol{k'}\in\text{BZ}} d^\dagger_{\text{BZ}(\text{BZ}(\boldsymbol{k}-\boldsymbol{q})+\boldsymbol{q}),\alpha} d_{\boldsymbol{k'},\delta} \mathcal{F}_{\alpha\beta}(\text{BZ}(\boldsymbol{k}-\boldsymbol{q}),\boldsymbol{q})\ \mathcal{F}_{\gamma\delta}(\boldsymbol{k'},-\boldsymbol{q})\ \delta_{\text{BZ}(\boldsymbol{k}-\boldsymbol{q}),\text{BZ}(\boldsymbol{k'}-\boldsymbol{q})}\delta_{\beta,\gamma} \\ &= \sum_{\boldsymbol{q}\notin\text{RL}} V(\boldsymbol{q}) \sum_{\boldsymbol{k}\in\text{BZ}} d^\dagger_{\boldsymbol{k}\alpha} d_{\boldsymbol{k}\delta} \mathcal{F}^\dagger_{\alpha\beta}(\boldsymbol{k},-\boldsymbol{q})\mathcal{F}_{\beta\delta}(\boldsymbol{k},-\boldsymbol{q}) \\ &= \sum_{\boldsymbol{q}\notin\text{RL}} V(\boldsymbol{q}) \sum_{\boldsymbol{k}\in\text{BZ}} d^\dagger_{\boldsymbol{k}} \mathcal{F}^\dagger(\boldsymbol{k},\boldsymbol{q})\mathcal{F}(\boldsymbol{k},\boldsymbol{q}) d_{\boldsymbol{k}} \\ &= \sum_{\boldsymbol{k}\in\text{BZ}} d^\dagger_{\boldsymbol{k}} h^{\text{single}}_1 d_{\boldsymbol{k}}, \quad \text{where} \quad h^{\text{single}}_1 = \sum_{\boldsymbol{q}\notin\text{RL}} V(\boldsymbol{q})\mathcal{F}^\dagger(\boldsymbol{k},\boldsymbol{q})\mathcal{F}(\boldsymbol{k},\boldsymbol{q})\ . \end{aligned} \tag{38}$$

This new term is included in the single-body term in Supplementary Equation (35) by replacing $\widetilde{h_{\boldsymbol{k}}} \to h_{\boldsymbol{k}} = \widetilde{h_{\boldsymbol{k}}} + h^{\text{single}}_1$.

Proceeding to the first term in Supplementary Equation (37) we first shift the index $\boldsymbol{k}' \to \mathrm{BZ}(\boldsymbol{k}'+\boldsymbol{q})$ and rearrange the annihilation operators as

$$\begin{aligned}\hat{H}_1^{\mathrm{eff}} &= -\sum_{\boldsymbol{q}\notin\mathrm{RL}} V(\boldsymbol{q}) \sum_{\boldsymbol{k},\boldsymbol{k}'\in\mathrm{BZ}} d^\dagger_{\mathrm{BZ}(\boldsymbol{k}+\boldsymbol{q}),\alpha} d^\dagger_{\boldsymbol{k}',\gamma} d_{\boldsymbol{k},\beta} d_{\mathrm{BZ}(\boldsymbol{k}'+\boldsymbol{q}),\delta} \mathcal{F}_{\alpha\beta}(\boldsymbol{k},\boldsymbol{q}) \mathcal{F}_{\gamma\delta}\left(\mathrm{BZ}(\boldsymbol{k}'+\boldsymbol{q}),-\boldsymbol{q}\right) \\ &= \sum_{\boldsymbol{q}\notin\mathrm{RL}} V(\boldsymbol{q}) \sum_{\boldsymbol{k},\boldsymbol{k}'\in\mathrm{BZ}} d^\dagger_{\mathrm{BZ}(\boldsymbol{k}+\boldsymbol{q}),\alpha} d^\dagger_{\boldsymbol{k}',\gamma} d_{\mathrm{BZ}(\boldsymbol{k}'+\boldsymbol{q}),\delta} d_{\boldsymbol{k},\beta} \mathcal{F}_{\alpha,\beta}(\boldsymbol{k},\boldsymbol{q}) \mathcal{F}^\dagger_{\gamma\delta}(\boldsymbol{k}',\boldsymbol{q}).\end{aligned} \tag{39}$$

After the mean-field decoupling,

$$\begin{aligned}&\hat{H}_1^{\mathrm{eff}} = \sum_{\boldsymbol{q}\notin\mathrm{RL}} V(\boldsymbol{q}) \sum_{\boldsymbol{k},\boldsymbol{k}'\in\mathrm{BZ}} \mathcal{F}_{\alpha\beta}(\boldsymbol{k},\boldsymbol{q}) \mathcal{F}^\dagger_{\gamma\delta}(\boldsymbol{k}',\boldsymbol{q}) \times \\ &\times \Big(\langle d^\dagger_{\boldsymbol{k}',\gamma} d_{\mathrm{BZ}(\boldsymbol{k}'+\boldsymbol{q}),\delta}\rangle\, d^\dagger_{\mathrm{BZ}(\boldsymbol{k}+\boldsymbol{q}),\alpha} d_{\boldsymbol{k},\beta} \delta_{\boldsymbol{k}',\mathrm{BZ}(\boldsymbol{k}'+\boldsymbol{q})} + \langle d^\dagger_{\mathrm{BZ}(\boldsymbol{k}+\boldsymbol{q}),\alpha} d_{\boldsymbol{k},\beta}\rangle\, d^\dagger_{\boldsymbol{k}',\gamma} d_{\mathrm{BZ}(\boldsymbol{k}'+\boldsymbol{q}),\delta} \delta_{\mathrm{BZ}(\boldsymbol{k}+\boldsymbol{q}),\boldsymbol{k}} + \\ &- \langle d^\dagger_{\mathrm{BZ}(\boldsymbol{k}+\boldsymbol{q}),\alpha} d_{\mathrm{BZ}(\boldsymbol{k}'+\boldsymbol{q}),\delta}\rangle\, d^\dagger_{\boldsymbol{k}',\gamma} d_{\boldsymbol{k},\beta} \delta_{\mathrm{BZ}(\boldsymbol{k}+\boldsymbol{q}),\mathrm{BZ}(\boldsymbol{k}'+\boldsymbol{q})} - \langle d^\dagger_{\boldsymbol{k}',\gamma} d_{\boldsymbol{k},\beta}\rangle\, d^\dagger_{\mathrm{BZ}(\boldsymbol{k}+\boldsymbol{q}),\alpha} d_{\mathrm{BZ}(\boldsymbol{k}'+\boldsymbol{q}),\delta} \delta_{\boldsymbol{k}',\boldsymbol{k}} \Big).\end{aligned} \tag{40}$$

The Hartree contribution is zero for the fermionic model, since $\delta_{\mathrm{BZ}(\boldsymbol{k}+\boldsymbol{q}),\boldsymbol{k}} = 0, \forall \boldsymbol{k} \in \mathrm{BZ}$ and $\boldsymbol{q} \notin \mathrm{RL}$. Therefore, with the Fock contribution as the only remaining part, the effective Hamiltonian takes the form

$$\hat{H}_1^{\mathrm{eff}} = -\sum_{\boldsymbol{q}\in\mathbb{R}} V(\boldsymbol{q}) \sum_{\boldsymbol{k}\in\mathrm{BZ}} \mathcal{F}_{\alpha\beta}(\boldsymbol{k},\boldsymbol{q}) \mathcal{F}^\dagger_{\gamma\delta}(\boldsymbol{k},\boldsymbol{q}) \left[(P_{\mathrm{BZ}(\boldsymbol{k}+\boldsymbol{q})})_{\alpha,\delta} d^\dagger_{\boldsymbol{k},\gamma} d_{\boldsymbol{k},\beta} + (P_{\boldsymbol{k}})_{\gamma,\beta} d^\dagger_{\mathrm{BZ}(\boldsymbol{k}+\boldsymbol{q}),\alpha} d_{\mathrm{BZ}(\boldsymbol{k}+\boldsymbol{q}),\delta} \right]$$

with the projector defined as $\left(P_{\mathrm{BZ}(\boldsymbol{k}+\boldsymbol{q})}\right)_{\alpha,\delta} = \langle d^\dagger_{\mathrm{BZ}(\boldsymbol{k}+\boldsymbol{q}),\alpha} d_{\mathrm{BZ}(\boldsymbol{k}'+\boldsymbol{q}),\delta}\rangle$. We also used the fact that $\delta_{\boldsymbol{k}',\boldsymbol{k}} = \delta_{\mathrm{BZ}(\boldsymbol{k}+\boldsymbol{q}),\mathrm{BZ}(\boldsymbol{k}'+\boldsymbol{q})}$. Switching back to matrix notation in

$$\hat{H}_1^{\mathrm{eff}} = -\sum_{\boldsymbol{q}\in\mathbb{R}} V(\boldsymbol{q}) \sum_{\boldsymbol{k}\in\mathrm{BZ}} \left[d^\dagger_{\boldsymbol{k}} \mathcal{F}^\dagger(\boldsymbol{k},\boldsymbol{q}) (P_{\mathrm{BZ}(\boldsymbol{k}+\boldsymbol{q})})^T \mathcal{F}(\boldsymbol{k},\boldsymbol{q})\ d_{\boldsymbol{k}} + d^\dagger_{\mathrm{BZ}(\boldsymbol{k}+\boldsymbol{q})} \mathcal{F}(\boldsymbol{k},\boldsymbol{q}) (P_{\boldsymbol{k}})^T \mathcal{F}^\dagger(\boldsymbol{k},\boldsymbol{q}) d_{\mathrm{BZ}(\boldsymbol{k}+\boldsymbol{q})} \right], \tag{41}$$

and taking another index shift in the second term $\boldsymbol{q} \to -\boldsymbol{q}$ and $\boldsymbol{k} \to \mathrm{BZ}(\boldsymbol{k}+\boldsymbol{q})$ yields

$$\hat{H}_1^{\mathrm{eff}} = -2 \sum_{\boldsymbol{q}\in\mathbb{R}} V(\boldsymbol{q}) \sum_{\boldsymbol{k}\in\mathrm{BZ}} d^\dagger_{\boldsymbol{k}} \left[\mathcal{F}^\dagger(\boldsymbol{k},\boldsymbol{q}) (P_{\mathrm{BZ}(\boldsymbol{k}+\boldsymbol{q})})^T \mathcal{F}((\boldsymbol{k},\boldsymbol{q}) \right] d_{\boldsymbol{k}}. \tag{42}$$

To summarize, within mean-field theory, we have found an effective Hamiltonian for the interacting term given by the Hatree-Fock equations

$$\begin{aligned}\hat{H}^{\mathrm{eff}} &= \sum_{\boldsymbol{k}\in\mathrm{BZ}} d^\dagger_{\boldsymbol{k}} (f[P_{\boldsymbol{k}}] + h_{\boldsymbol{k}}) d_{\boldsymbol{k}}, \\ f[P_{\boldsymbol{k}}] &= -2 \sum_{\boldsymbol{q}\notin\mathrm{RL}} V(\boldsymbol{q}) \left(\mathcal{F}^\dagger(\boldsymbol{k},\boldsymbol{q}) (P_{\mathrm{BZ}(\boldsymbol{k}+\boldsymbol{q})})^T \mathcal{F}(\boldsymbol{k},\boldsymbol{q})\ \right), \\ h_{\boldsymbol{k}} &= \sum_{\boldsymbol{q}\notin\mathrm{RL}} V(\boldsymbol{q}) \mathcal{F}^\dagger(\boldsymbol{k},\boldsymbol{q}) \mathcal{F}(\boldsymbol{k},\boldsymbol{q})\ + t\cos(\boldsymbol{k})\sigma_x.\end{aligned} \tag{43}$$

which needs to be solved self-consistently. The HF ground state energy is then given by

$$E_{\mathrm{HF}} = \sum_{\boldsymbol{k}\in\mathrm{BZ}} \left((f[P_{\boldsymbol{k}}] + h_{\boldsymbol{k}})_{-,-} - E^0_{\boldsymbol{k}} \right) \tag{44}$$

with the energy offset

$$E^0_{\boldsymbol{k}} = \frac{1}{2} \mathrm{Tr}\left[P^T_{\boldsymbol{k}}\right] f[P_{\boldsymbol{k}}] = -\sum_{\boldsymbol{q}\notin\mathrm{RL}} V(\boldsymbol{q})\, \mathrm{Tr}\left[P^T_{\boldsymbol{k}} \mathcal{F}^\dagger(\boldsymbol{k},\boldsymbol{q}) (P_{\mathrm{BZ}(\boldsymbol{k}+\boldsymbol{q})})^T \mathcal{F}(\boldsymbol{k},\boldsymbol{q}) \right]. \tag{45}$$

To solve Supplementary Equation (43), we first consider an initial ansatz for P. This can be chosen to correspond to the (i) the interacting limit $t/U \to 0$ with $P^{\mathrm{chiral}}_{\boldsymbol{k}} = \mathrm{diag}\,(0,1)$, (ii) the kinetic edge case $P^{\mathrm{band}}_{\boldsymbol{k}} = (\sigma_0 - \sigma_x)/2$ or (iii) to a random matrix $P^{\mathrm{rand}}_{\boldsymbol{k}}$ that fulfills the projector properties $P_{\boldsymbol{k}} = P^\dagger_{\boldsymbol{k}} = (P_{\boldsymbol{k}})^2$. In all simulations for this work we always choose (iii).

The Hermitian matrix $(f[P_{\boldsymbol{k}}]+h_{\boldsymbol{k}})$ in Supplementary Equation (43) is diagonalized in each iteration step according to

$$D_{\boldsymbol{k}} = U_{\boldsymbol{k}}^{-1}(f[P_{\boldsymbol{k}}]+h_{\boldsymbol{k}})U_{\boldsymbol{k}}, \quad D_{\boldsymbol{k}} = \begin{pmatrix} E_{\boldsymbol{k}}^{+} & 0 \\ 0 & E_{\boldsymbol{k}}^{-} \end{pmatrix} \quad \text{and} \quad U_k = \begin{pmatrix} v_0^{+} & v_0^{-} \\ v_1^{+} & v_1^{-} \end{pmatrix}, \tag{46}$$

where we ensure that $E_{\boldsymbol{k}}^{-} \leq E_{\boldsymbol{k}}^{+}$ in the $\boldsymbol{k}$-dependent diagonal matrix $D_{\boldsymbol{k}}$, and the unitary matrix $U_{\boldsymbol{k}}$ is composed of the normalized eigenvectors v^{+} and v^{-}. We initialize $D_{\boldsymbol{k}} = \mathrm{diag}\,(0,1)$, which corresponds to filling the lower energy band of the mean-field Hamiltonian $\hat{H}_{\mathrm{HF}}$, and calculate the projector of the next iteration step $P_{\boldsymbol{k}}^{i+1} = U_{\boldsymbol{k}}^{*} D U_{\boldsymbol{k}}^{T}$ which, in turn, defines $f[P_{\boldsymbol{k}}^{i+1}]$. This is done until the projector converges according to $\|P_{\boldsymbol{k}}^{(i+1)} - P_{\boldsymbol{k}}^{i}\|_F < 10^{-10}$ where $\|\cdot\|_F$ denotes the Frobenius norm.

After convergence we can also evaluate other observables besides the ground state energy, like the momentum-resolved fermionic bilinears according to Equation (11). Within HF, in Supplementary Fig. 1a-c we show the band structure ($\epsilon_{\boldsymbol{k},p}$ in Equation (3)) and the fermionic bilinears in the chiral basis (d), which illustrate the metal-insulator phase transition described in the main text. For small t/U we see a clear band gap in the insulating regime (see Supplementary Fig. 1a). The energy gap gets smaller for intermediate t/U, and vanishes for large t/U (c) with the appearance of two Dirac cones around $|\boldsymbol{k}| = \pm\pi/2$. By defining the order parameter $\xi = \sum_{\boldsymbol{k}} \mathcal{N}_{\boldsymbol{k}}^{z}/N_e$, we see that the metal-insulator phase transition is set around $t = 0.14$ (c). From Supplementary Fig. 1d, we also note that at $t/U = 0$, $\langle \mathcal{N}_{\boldsymbol{k}}^{x} \rangle = 0$ while large t/U leads to $|\langle \mathcal{N}_{\boldsymbol{k}}^{x} \rangle| = 1$ with sign changes at $|\boldsymbol{k}| = \pm\pi/2$.

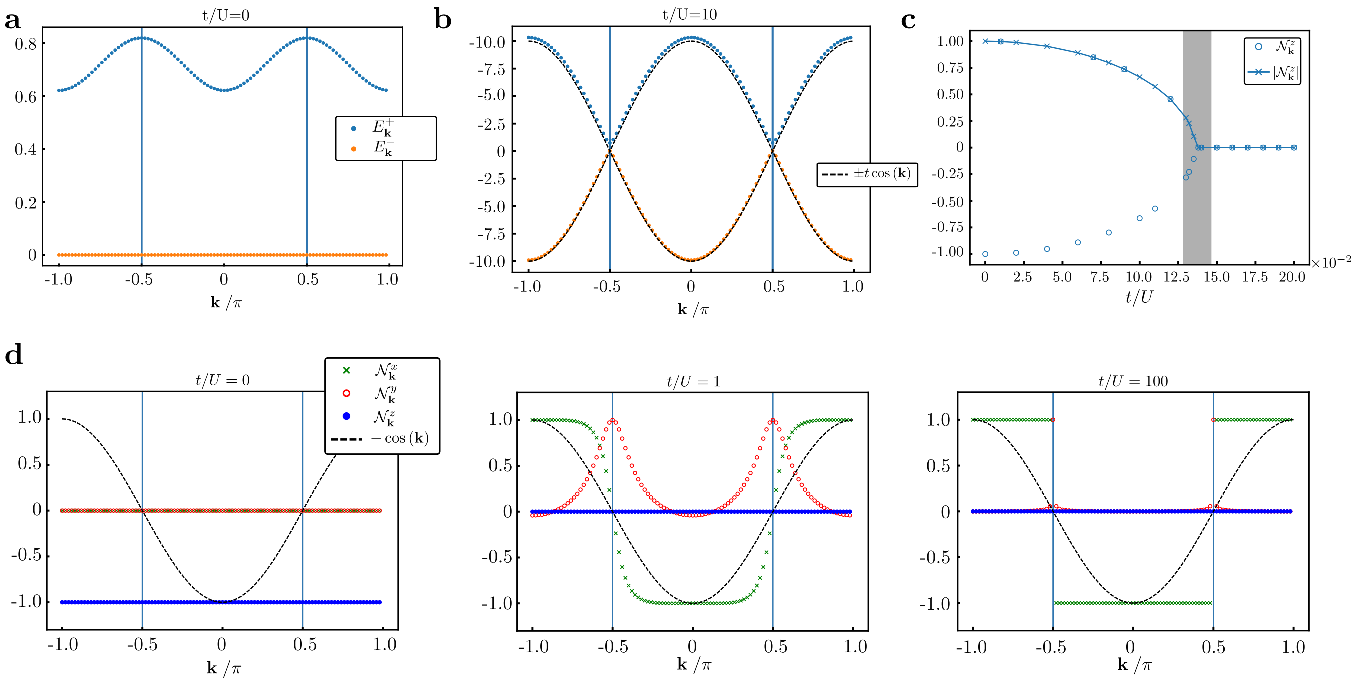

Supplementary Fig. 1 | Observables for the fermionic model from the perspective of Hartree-Fock for $N_e = 100$ electrons. Band structure for the fermionic model at $t/U = 0$ (**a**) and $t/U = 10$ (**b**). **c** Order parameter ξ as a function of t/U. **d** Momentum-resolved fermionic bilinears (according to Equation (11)) as a function of $\boldsymbol{k}$ for different values of t/U.

SUPPLEMENTARY NOTE B: VARIATIONAL MONTE CARLO

5. Calculating observables

According to the ansatz Equation (4), the expectation value

$$\frac{\langle\Psi_{\{\boldsymbol{\theta},\alpha\}}|\hat{O}|\Psi_{\{\boldsymbol{\theta},\alpha\}}\rangle}{\langle\Psi_{\{\boldsymbol{\theta},\alpha\}}\mid\Psi_{\{\boldsymbol{\theta},\alpha\}}\rangle} = \frac{1}{\alpha^2+(1-\alpha^2)\sum_{\boldsymbol{s}''\neq\mathrm{RS}}|\psi_{\boldsymbol{\theta}}(\boldsymbol{s}'')|^2}\Bigg[\alpha^2\langle\mathrm{RS}|\,\hat{O}\,|\mathrm{RS}\rangle+\alpha\sqrt{1-\alpha^2}\sum_{\boldsymbol{s}\neq\mathrm{RS}}\langle\mathrm{RS}|\hat{O}|\boldsymbol{s}\rangle\,\psi_{\boldsymbol{\theta}}(\boldsymbol{s})+$$
$$+\sum_{\boldsymbol{s}\neq\mathrm{RS}}\left(\left(1-\alpha^2\right)\sum_{\boldsymbol{s}'\neq\mathrm{RS}}\langle\boldsymbol{s}|\hat{O}|\boldsymbol{s}'\rangle\,\psi_{\boldsymbol{\theta}}(\boldsymbol{s}')\,\psi^*_{\boldsymbol{\theta}}(\boldsymbol{s})+\alpha\sqrt{1-\alpha^2}\,\langle\boldsymbol{s}|\hat{O}|\mathrm{RS}\rangle\,\psi^*_{\boldsymbol{\theta}}(\boldsymbol{s})\right)\Bigg]$$
$$=\frac{1}{\mathbb{N}_{\{\boldsymbol{\theta},\alpha\}}}\left[\alpha^2 O_{\mathrm{RS}}+\left(1-\alpha^2\right)\sum_{\boldsymbol{s},\boldsymbol{s}'\neq\mathrm{RS}}|\psi_{\boldsymbol{\theta}}(\boldsymbol{s})|^2\langle\boldsymbol{s}|\hat{O}|\boldsymbol{s}'\rangle\frac{\psi_{\boldsymbol{\theta}}(\boldsymbol{s}')}{\psi_{\boldsymbol{\theta}}(\boldsymbol{s})}+\alpha\sqrt{1-\alpha^2}\sum_{\boldsymbol{s}\neq\mathrm{RS}}|\psi_{\boldsymbol{\theta}}(\boldsymbol{s})|^2\left(O_{\boldsymbol{s}\mathrm{RS}}\frac{1}{\psi_{\boldsymbol{\theta}}(\boldsymbol{s})}+O_{\mathrm{RS}\boldsymbol{s}}\frac{1}{\psi^*_{\boldsymbol{\theta}}(\boldsymbol{s})}\right)\right]$$
$$=\frac{1}{\mathbb{N}_{\{\boldsymbol{\theta},\alpha\}}}\left[\alpha^2 O_{\mathrm{RS}}+\left(1-\alpha^2\right)\sum_{\boldsymbol{s}\neq\mathrm{RS}}|\psi_{\boldsymbol{\theta}}(\boldsymbol{s})|^2 O^{\boldsymbol{s}'\neq\mathrm{RS}}_{\mathrm{loc}}(\boldsymbol{s})+\alpha\sqrt{1-\alpha^2}2\mathrm{Re}\left(\sum_{\boldsymbol{s}\neq\mathrm{RS}}|\psi_{\boldsymbol{\theta}}(\boldsymbol{s})|^2 O_{\boldsymbol{s}\mathrm{RS}}\frac{1}{\psi_{\boldsymbol{\theta}}(\boldsymbol{s})}\right)\right], \tag{47}$$

which is equivalent to Equation (14) for the expectation value of $\hat{O}=\hat{H}$. Note that the normalization factor $\mathbb{N}_{\{\boldsymbol{\theta},\alpha\}}=\alpha^2+\left(1-\alpha^2\right)\sum_{\boldsymbol{s}''\neq\mathrm{RS}}|\psi_{\boldsymbol{\theta}}(\boldsymbol{s}'')|^2\to 1$ for any α if the RS is not sampled from the TQS. Similarly, for the gradient of the energy functional

$$\nabla_{\boldsymbol{\theta}}E(\boldsymbol{\theta},\alpha)=\partial_{\boldsymbol{\theta}}\left[\sum_{\boldsymbol{s},\boldsymbol{s}'\neq\mathrm{HF}}\left(1-\alpha^2\right)\psi^*_{\boldsymbol{\theta}}(\boldsymbol{s})\,H_{\boldsymbol{s}\boldsymbol{s}'}\psi_{\boldsymbol{\theta}}(\boldsymbol{s}')+\alpha\sqrt{1-\alpha^2}\sum_{\boldsymbol{s}\neq\mathrm{RS}}\left(\psi^*_{\boldsymbol{\theta}}(\boldsymbol{s})\,H_{\boldsymbol{s}\mathrm{RS}}+H_{\mathrm{RS}\boldsymbol{s}}\psi_{\boldsymbol{\theta}}(\boldsymbol{s})\right)\right]$$
$$=\left(1-\alpha^2\right)\sum_{\boldsymbol{s},\boldsymbol{s}'\neq\mathrm{HF}}\left(\partial_{\boldsymbol{\theta}}\psi^*_{\boldsymbol{\theta}}(\boldsymbol{s})\,\psi_{\boldsymbol{\theta}}(\boldsymbol{s}')\frac{\psi^*_{\boldsymbol{\theta}}(\boldsymbol{s})}{\psi^*_{\boldsymbol{\theta}}(\boldsymbol{s})}\frac{\psi_{\boldsymbol{\theta}}(\boldsymbol{s})}{\psi_{\boldsymbol{\theta}}(\boldsymbol{s})}+\psi^*_{\boldsymbol{\theta}}(\boldsymbol{s})\,\partial_{\boldsymbol{\theta}}\psi_{\boldsymbol{\theta}}(\boldsymbol{s}')\frac{\psi^*_{\boldsymbol{\theta}}(\boldsymbol{s}')}{\psi^*_{\boldsymbol{\theta}}(\boldsymbol{s}')}\frac{\psi_{\boldsymbol{\theta}}(\boldsymbol{s}')}{\psi_{\boldsymbol{\theta}}(\boldsymbol{s}')}\right)H_{\boldsymbol{s}\boldsymbol{s}'}+$$
$$+\alpha\sqrt{1-\alpha^2}\sum_{\boldsymbol{s}\neq\mathrm{RS}}\left(\partial_{\boldsymbol{\theta}}\psi^*_{\boldsymbol{\theta}}(\boldsymbol{s})\,H_{\boldsymbol{s}\mathrm{RS}}\frac{\psi_{\boldsymbol{\theta}}(\boldsymbol{s})}{\psi_{\boldsymbol{\theta}}(\boldsymbol{s})}\frac{\psi^*_{\boldsymbol{\theta}}(\boldsymbol{s})}{\psi^*_{\boldsymbol{\theta}}(\boldsymbol{s})}+H_{\mathrm{RS}\boldsymbol{s}}\partial_{\boldsymbol{\theta}}\psi_{\boldsymbol{\theta}}(\boldsymbol{s})\frac{\psi_{\boldsymbol{\theta}}(\boldsymbol{s})}{\psi_{\boldsymbol{\theta}}(\boldsymbol{s})}\right)$$
$$=\left(1-\alpha^2\right)2\mathrm{Re}\left\langle\partial_{\boldsymbol{\theta}}\log\psi^*_{\boldsymbol{\theta}}(\boldsymbol{s})\,H^{\boldsymbol{s}'\neq\mathrm{RS}}_{\mathrm{loc}}(\boldsymbol{s})\right\rangle+ \tag{48}$$
$$+\alpha\sqrt{1-\alpha^2}\left(\left\langle\partial_{\boldsymbol{\theta}}\log\psi^*_{\boldsymbol{\theta}}(\boldsymbol{s})\,H_{\boldsymbol{s}\mathrm{RS}}\frac{1}{\psi_{\boldsymbol{\theta}}(\boldsymbol{s})}\right\rangle+\sum_{\boldsymbol{s}\neq\mathrm{RS}}\partial_{\boldsymbol{\theta}}\log\psi_{\boldsymbol{\theta}}(\boldsymbol{s})\,H_{\mathrm{RS}\boldsymbol{s}}\psi_{\boldsymbol{\theta}}(\boldsymbol{s})\right),$$
$$=\left(1-\alpha^2\right)2\mathrm{Re}\left\langle\partial_{\boldsymbol{\theta}}\log\psi^*_{\boldsymbol{\theta}}(\boldsymbol{s})\,H^{\boldsymbol{s}'\neq\mathrm{RS}}_{\mathrm{loc}}(\boldsymbol{s})\right\rangle+\alpha\sqrt{1-\alpha^2}2\mathrm{Re}\left\langle\partial_{\boldsymbol{\theta}}\log\psi^*_{\boldsymbol{\theta}}(\boldsymbol{s})\,H_{\boldsymbol{s}\mathrm{RS}}\frac{1}{\psi_{\boldsymbol{\theta}}(\boldsymbol{s})}\right\rangle,$$
$$=2\mathrm{Re}\left[\left\langle\partial_{\boldsymbol{\theta}}\log\psi^*_{\boldsymbol{\theta}}(\boldsymbol{s})\left(\left(1-\alpha^2\right)H^{\boldsymbol{s}'\neq\mathrm{RS}}_{\mathrm{loc}}(\boldsymbol{s})+\alpha\sqrt{1-\alpha^2}H_{\boldsymbol{s}\mathrm{RS}}\frac{1}{\psi_{\boldsymbol{\theta}}(\boldsymbol{s})}\right)\right\rangle\right],$$

in accordance with Equation (15) from the main text.

6. Performance analysis of optimizers and hyperparameters

The main hyperparameters that define a decoder-only Transformer architecture are given by N_{dec}, d_{emb} and, N_{h} which stand, respectively, for the number of encoding layers, embedding dimension and number of attention heads (see Fig. 1b). For a more in-depth description of this architecture, we refer the reader to Vaswani et al and Lin et al.[2,3] More importantly, the representational power of these architectures is directly related to these quantities.[4] For the tree sampler in Fig. 1c we always used $N_s=1\times10^8$ and $n_U=4\times10^3$, unless stated otherwise. In more general scenarios one can also adopt a data-driven criterion for choosing n_U: when computationally feasible, one can set n_U based on convergence trends observed in smaller system sizes as a function of t/U. However, when hardware limitations restrict access to sufficiently large n_U values, the accuracy of the ground state estimate becomes bounded by the number of states sampled in n_U, as illustrated in Fig. 2a of the main text.

In Supplementary Fig. 2 we show how the training of the Transformers with the HF-basis is affected in terms of different combinations of these parameters. We focus on $N_e = 10$ electrons, but analogous results can be directly obtained for larger system sizes. For $t/U = 0.10$, we see that training can be made faster and more accurate by increasing only d_{emb}, or, if kept fixed, by increasing N_{dec} and N_h. Similarly, we observed the same behavior for $t/U \in [0.01, 0.04] \cup [0.10, 0.20]$. Therefore, we used the combination $d_{\text{emb}} = 300$, $N_{\text{dec}} = 4$ and $N_h = 10$ for the plots in Fig. 2a and Fig. 3 of the main text. Additionally, the results in Fig. 2(a,d) were obtained with $n_{\text{epochs}} = 2 \times 10^4$.

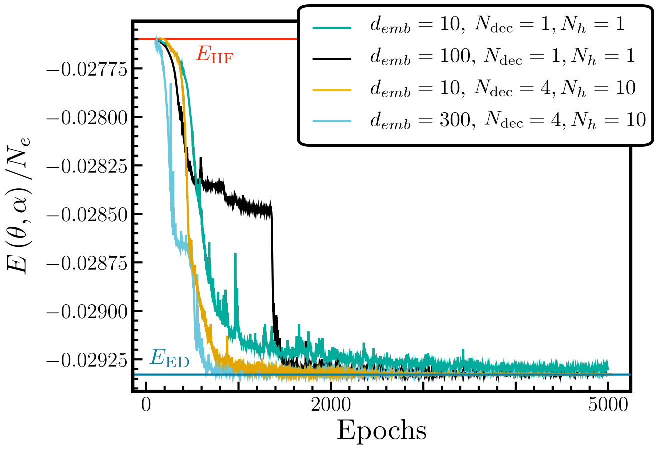

Supplementary Fig. 2 | HF-TQS performance with distinct hyperparameters. Ground state energy per electron as a function of epochs for distinct combinations of d_{emb}, N_{dec} and N_h at $t/U = 0.10$ for $N_e = 10$. The dashed blue (red) line indicates the ground state energy obtained from ED (HF).

For the optimization of the $\boldsymbol{\theta}$ parameters, the learning rate $\lambda_{\boldsymbol{\theta}}$ is changed according to the following scheduler[5]

$$\lambda_{\boldsymbol{\theta}}^{i+1} = \lambda_{\boldsymbol{\theta}}^{i} + \beta d_{\text{emb}}^{-0.5} \min\left(i_{\text{step}}^{-0.75}, i_{\text{step}}\, i_{\text{warmup}}^{-1.75}\right) \tag{49}$$

with $\beta = 0.2$ representing a scale factor, i_{step} the current iteration index, and $i_{\text{warmup}} = 700$ the number of warming up steps. This scheduler allows the learning rate to be increased linearly during the first i_{warmup} epochs and then decreased polynomially during the remaining ($n_{\text{epochs}} - i_{warmup}$) steps.[2] Although a similar learning rate scheduler can be imposed for the optimization of α according to Equation (16), we noticed that the Transformer converges better for $t/U < 0.10$ with a constant learning rate. The best converged energies between $\lambda_{\alpha_0} = 100$ (for intermediate t/U) and $\lambda_{\alpha_0} = 2$ (for small t/U) are shown for the simulations in Fig. 2 and Fig. 3 of the main text.

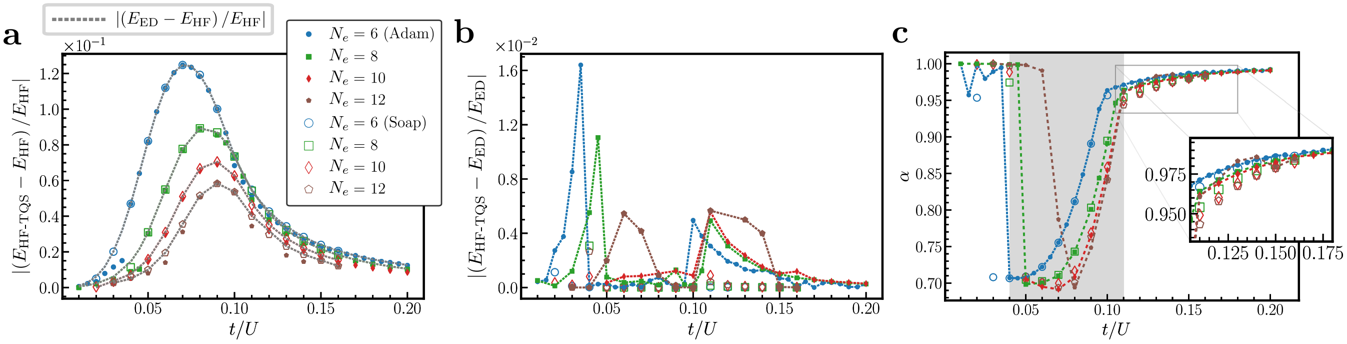

Supplementary Fig. 3 | HF-TQS results with ADAM and SOAP optimizers. Comparison of HF-TQS performance using ADAM[6] (filled markers) and SOAP[8] (unfilled markers). **a** Relative error $|(E_{\text{HF-TQS}} - E_{\text{HF}})/E_{\text{HF}}|$ as a function of t/U for various system sizes N_e. Dashed lines show the corresponding error between ED and HF. **b** Relative error $|(E_{\text{HF-TQS}} - E_{\text{ED}})/E_{\text{ED}}|$ as a function of t/U. **c** Corresponding converged α values according to Equation (14). The gray region indicates the metal-insulator transition vicinity. Dashed lines in panels **b** and **c** guide the eye.

Most importantly, we noticed that the choice of the specific stochastic optimizer was the most important point for a smooth and consistent convergence for different values of t/U for the fermionic model. More specifically, adaptive

moment estimation (ADAM)[6] and other variations of the simple stochastic gradient descent method tended to converge to ground state energies with lower accuracy away from the critical region (see Supplementary Fig. 3), irrespective of different combinations of the previously mentioned hyperparameters. We noticed a significant improvement when considering preconditioned gradient methods[7,8] in these regions. Similarly, there are also deviations for the converged α using these different optimizers, especially around the borders of the gray region in Supplementary Fig. 3c.

7. Further results: Larger system sizes, n_U scaling at the critical region and locality

In this section we present additional results to complement the discussions in the main text. Supplementary Fig. 4a

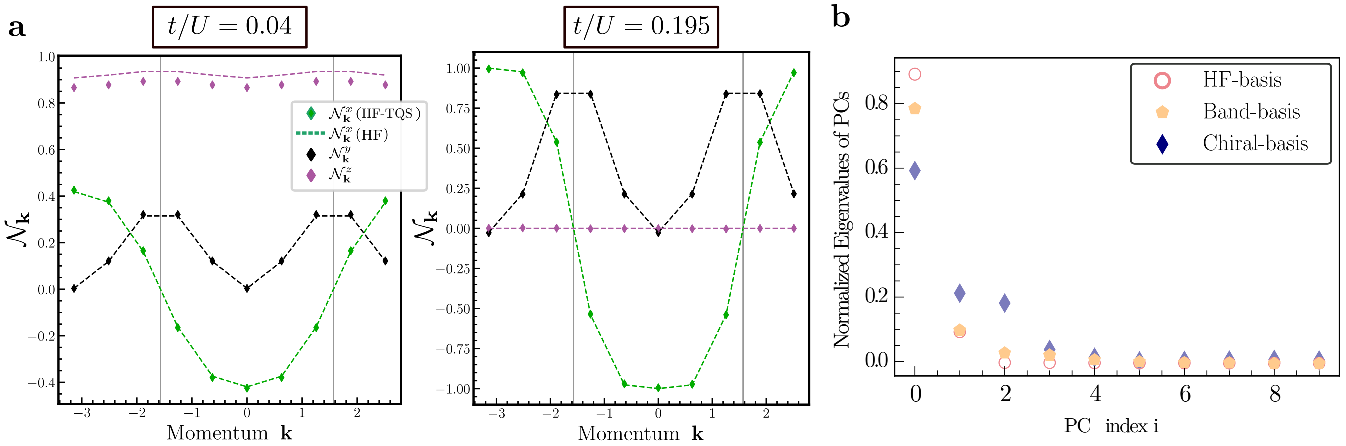

Supplementary Fig. 4 | Additional results for fermionic bilinear operators and PCA. a HF-TQS results (markers) for the momentum-resolved fermionic bilinears $\mathcal{N}^j_{\boldsymbol{k}}$, defined in Equation (11), compared with HF (dashed lines) for $N_e = 10$ at $t/U = 0.04$ (left) and $t/U = 0.195$ (right). **b** Normalized eigenvalues of the first ten principal components (PC) from the PCA analysis shown in Fig. 5 ($t/U = 0.12$) for the three distinct bases (indicated by markers) defined in the main text.

demonstrates that corrections to HF for the expectation values of the fermionic bilinears, cf. Equation (11), diminish away from the critical region, in accordance with the converged values for α in Fig. 3b.

Our PCA analysis of the latent space, shown in Fig. 5 of the main text, reveals that the first two principal components account for at least 80% of the total data variation across all bases (see Supplementary Fig. 4b). This high percentage of explained variance remained consistent, with slight variations, for other coupling parameters such as $t/U = 0.04$ and $t/U = 0.16$ in Fig. 5.

We also examined the Transformer's convergence in the band basis for large t/U. As shown in Supplementary Fig. 5, even though the fully polarized RS is not a good representative of the ground state in this regime, the Transformer is still able to achieve good agreement with ED, as stated in the main text. Furthermore, from Supplementary Fig. 5(c), we see that even if one of the states of the class $\mathcal{E}(\boldsymbol{s}) = 5$ were chosen as a reference state, since they are responsible for $\sim 25\%$ of the statistical weight (cf. Equation (10)), the remaining classes would still contribute to $\sim 75\%$ of the total ground state energy in this regime. This should be contrasted once more with Fig. 3c, where the ground state energy would be accurately computed with HF as an effective theory by only focusing on the double particle-hole excitations sector $\mathcal{E}(\boldsymbol{s}) = 2$.

In the critical region, we highlight how the performance of the HF-TQS depends on n_U. In Supplementary Fig. 6, we show an example with larger n_U for $N_e = 14$ at $t/U \simeq 0.10$ to demonstrate this point. Since $n_U > 2^{14} > n_U^{\text{previous}} = 4000$, more corrections are captured and α deviates more from unity near the phase transition – see white dots in Fig 2a of the main text. The histogram in Supplementary Fig. 6c should be compared with the one at $t/U = 0.09$ in Fig. 3c. The final number of states n_U^f retained by the Transformer is very close to 2^{14}. Importantly, the hierarchy over particle-hole excitation classes $\mathcal{E}(\mathbf{s})$ is preserved, in contrast to its absence in other effective theories $\hat{H}_0$, with double particle-hole excitations still dominating the corrections. For consistency, similar results should hold for $N_e \geq 14$ in the gray region of Fig. 2a.

Away from the critical regime, there is still a substantial advantage when considering HF as an effective theory, since $n_U^f \ll 2^{N_e}$ for any system size N_e, which we highlight in Supplementary Fig. 7 for $N_e = 60$ electrons at

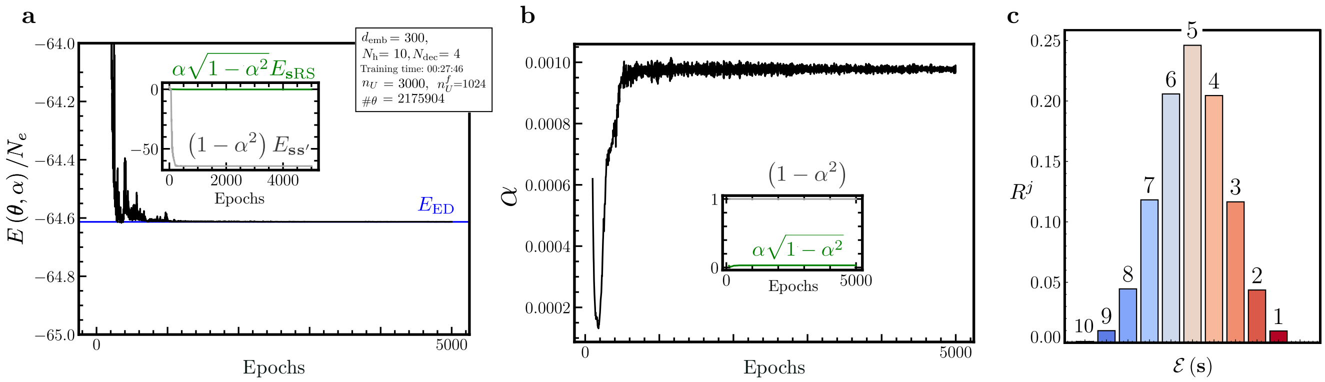

Supplementary Fig. 5 | TQS in the band basis for large t/U. Convergence of the ground state energy per electron (**a**) and the corresponding α weights (**b**) as a function of epochs for $N_e = 10$ and $t/U = 100$. (**c**) Histogram indicating the total relative frequencies R^j, according to Equation (10), for the excitation classes $\mathcal{E}(\mathbf{s})$ from Equation (9).

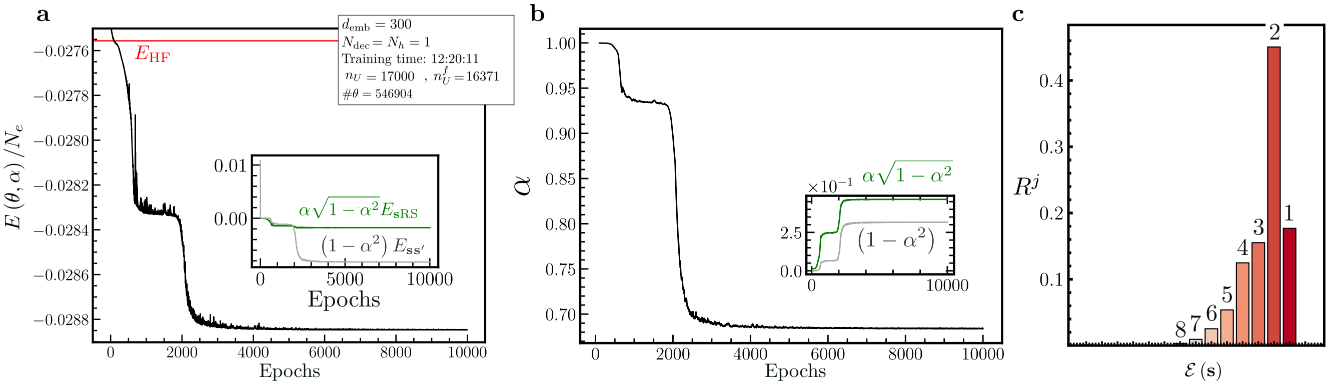

Supplementary Fig. 6 | HF-TQS for $N_e = 14$ at $t/U = 0.10$ and $n_U = 17000$. Convergence of the ground state energy per electron (**a**) and the corresponding α weights (**b**) as a function of epochs. (**c**) Histogram indicating the total relative frequencies R^j, according to Equation (10), for the excitation classes $\mathcal{E}(\mathbf{s})$ from Equation (9).

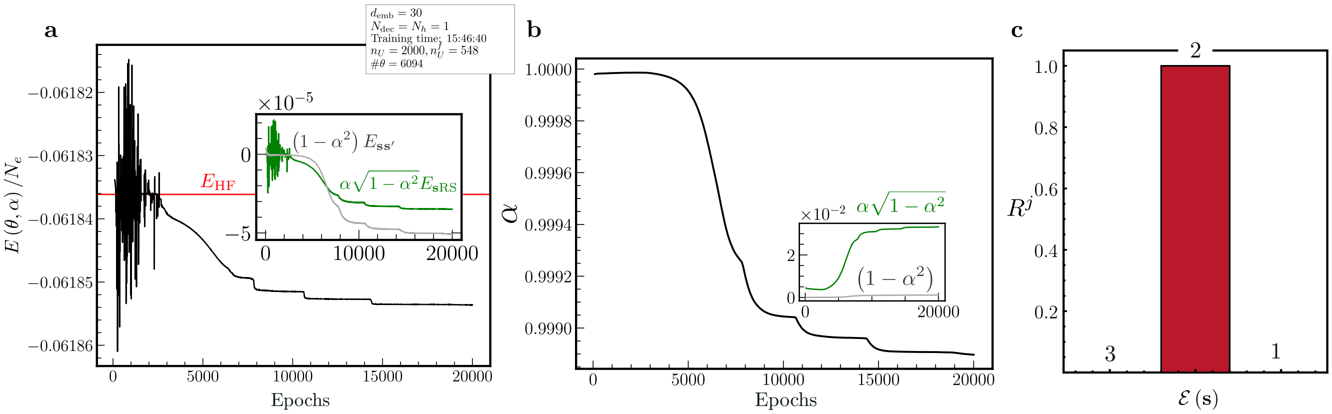

Supplementary Fig. 7 | HF-TQS for $N_e = 60$ at $t/U = 0.16$. Convergence of the ground state energy per electron (**a**) and the corresponding α weights (**b**) as a function of epochs. (**c**) Histogram indicating the total relative frequencies R^j, according to Equation (10), for the excitation classes $\mathcal{E}(\mathbf{s})$ from Equation (9).

$t/U = 0.16$. From the histogram, one can see that the number of final unique states is bounded by $n_U^f < C(60, 2)$, where $C(N_e, k) = \frac{N_e!}{k!(N_e-k)!}$. We then confirm that the sampling advantage for HF-TQS observed for smaller system sizes in the metallic regime (see Fig. 2 and Fig. 3) is also observed for larger system sizes. This result should be compared with the weak (band) and strong (chiral) coupling limits, where achieving the same ground state energy accuracy would still require a number of corrections that scales exponentially with system size, in accordance with Fig. 3c.

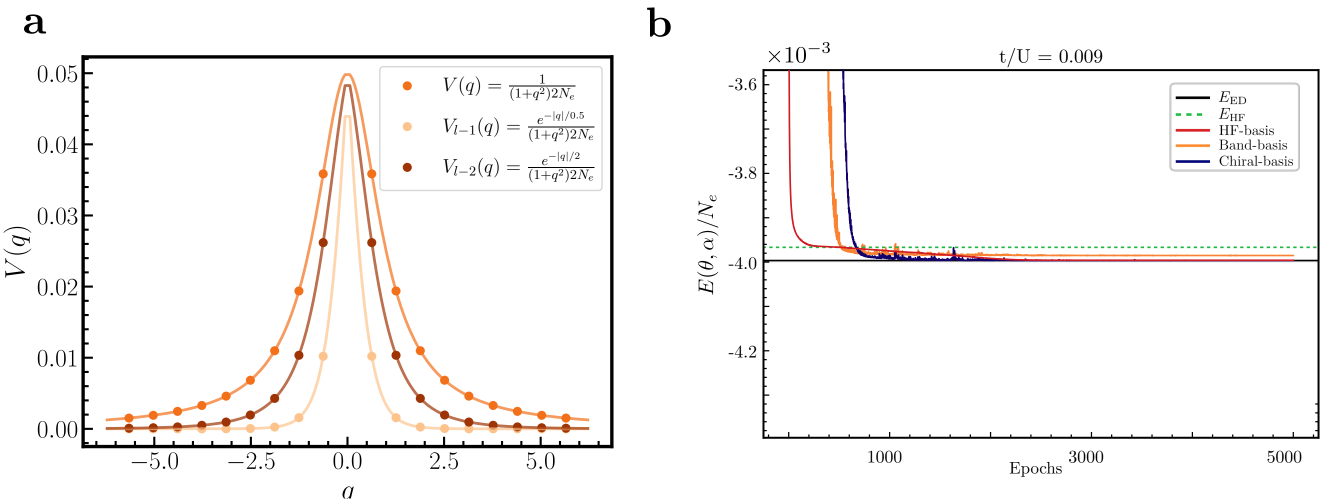

Supplementary Fig. 8 | Localized potentials. **a** Previous Coulomb interaction in comparison to localized modifications. Dots represent accessible scattering vectors q for $N_e = 10$. The potential $V_{l-1}(q)$ is effectively a first-neighbor-only interaction for this system size. **b** Ground state energy per electron as a function of epochs in the band, HF and chiral bases at $t/U = 0.009$ with the $V_{l-1}(q)$ potential.

Locality can be tuned in this model by modifying the Coulomb interaction (see Supplementary Fig. 8**a**) in momentum space via $V(q) = \exp(-|q|/\lambda)/(1 + q^2)$. Here, the parameter λ controls the degree of locality. For instance, the case $\lambda = 2$ (denoted $V_{l-2}(q)$) ensures that the potential magnitudes for first neighbors are comparable to those of the original Coulomb potential, while $\lambda = 0.5$ (denoted $V_{l-1}(q)$) defines an effective first-neighbor-only potential.

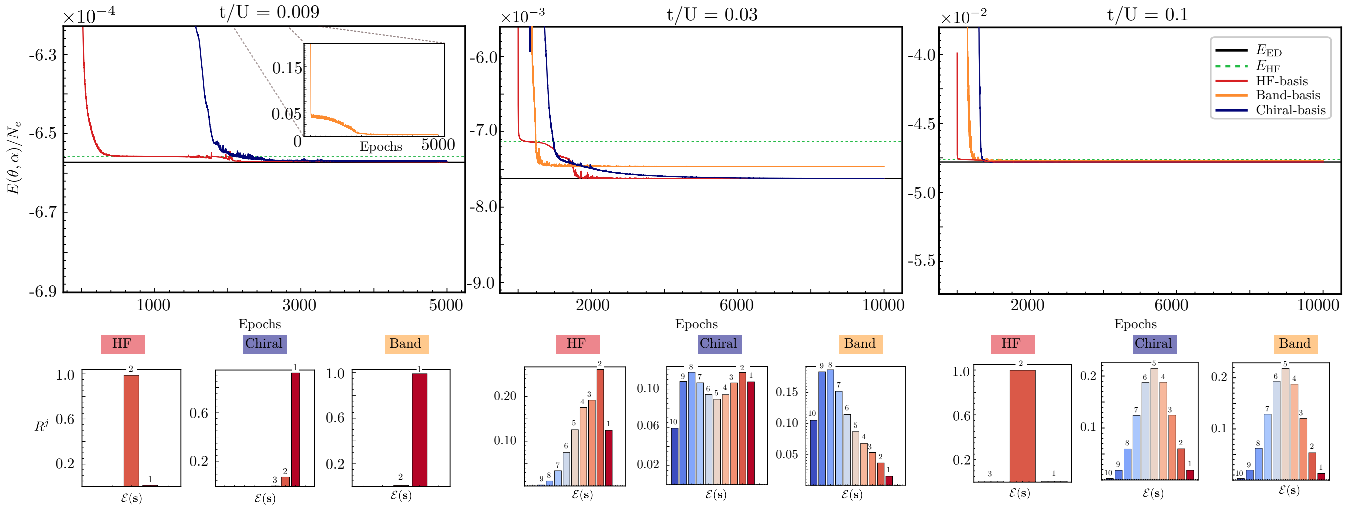

Supplementary Fig. 9 | Simulations with the $V_{l-2}(q)$ potential. Convergence of the ground state energy $E(\theta, \alpha)/N_e$ in the band, HF and chiral bases as a function of epochs for $t/U = 0.009$ (insulating) and $t/U = 0.03$ (critical) and $t/U = 0.1$ (metallic). All for $N_e = 10$. The histograms below each plot indicate the total relative frequencies R^j, for the excitation classes $\mathcal{E}(\mathbf{s})$.

For comparison with ED, we focused on $N_e = 10$ electrons for the following simulations. For different values of λ, we noted that the critical point was pushed to smaller coupling constants t/U, and thus the insulating regime in the phase diagram is much narrower. Starting from the metallic side ($t/U = 0.1$) for the potential $V_{l-2}(q)$, we have the same behavior as before, i.e., the "best basis" (with respect to accuracy and smaller n_U^f) is still HF, with double particle-hole excitations dominating the corrections, as can be seen from the plots in Supplementary Fig. 9. For $t/U = 0.03$, we again see the weights being distributed among all states in the Hilbert space for all bases. Finally, for $t/U = 0.009$, we observed that the band basis continues to perform quite poorly in the insulating regime, as can be seen from the inset of $E(\theta, \alpha)/N_e$ vs. epochs, converging to a higher energy estimate $E_{\text{band}} \to 0$. We have also observed that the HF basis needs a more frequent preconditioner update (5 instead of 50, which was used to get the results in Supplementary Fig. 3)[8] for more stable convergence in this regime.

Finally, we have also noticed that the effect of constraining the potential to first neighbors only—see $V_{l-1}(q)$ in Supplementary Fig. 8—just pushes the insulator to even smaller t/U coupling parameters. In Supplementary Fig. 8**b**, we show that the Transformer is still able to find corrections beyond HF for all three bases in this local version of the model for $t = 0.009$.

Supplementary References

[1] Christos, M., Sachdev, S. & Scheurer, M. S. Correlated insulators, semimetals, and superconductivity in twisted trilayer graphene. *Phys. Rev. X* **12**, 021018 (2022).

[2] Vaswani, A. et al. Attention is all you need. Preprint at https://doi.org/10.48550/arXiv.1706.03762 (2017).

[3] Lin, T., Wang, Y., Liu, X. & Qiu, X. A survey of transformers. Preprint at https://doi.org/10.48550/arXiv.2106.04554 (2021).

[4] Sanford, C., Hsu, D. & Telgarsky, M. Representational strengths and limitations of transformers. Preprint at https://doi.org/10.48550/arXiv.2306.02896 (2023).

[5] Zhang, Y.-H. & Di Ventra, M. Transformer quantum state: A multipurpose model for quantum many-body problems. *Phys. Rev. B* **107**, 075147 (2023).

[6] Kingma, D. P. & Ba, J. Adam: A method for stochastic optimization. Preprint at https://doi.org/10.48550/arXiv.1412.6980 (2014).

[7] Gupta, V., Koren, T. & Singer, Y. Shampoo: Preconditioned stochastic tensor optimization. Preprint at https://doi.org/10.48550/arXiv.1802.09568 (2018).

[8] Vyas, N. et al. SOAP: Improving and stabilizing shampoo using Adam. Preprint at https://doi.org/10.48550/arXiv.2409.11321 (2024).
